# New records of amphibians from Ha Tinh Province, Vietnam

**DOI:** 10.3897/BDJ.12.e122598

**Published:** 2024-05-08

**Authors:** Vinh Quang Dau, Cuong The Pham, Truong Quang Nguyen, Toan Canh Thai, Anh Dinh Tran, Anh Van Pham

**Affiliations:** 1 Hong Duc University, 565 Quang Trung Road, Dong Ve Ward, Thanh Hoa City, Vietnam Hong Duc University, 565 Quang Trung Road, Dong Ve Ward Thanh Hoa City Vietnam; 2 Institute of Ecology and Biological Resources and Graduate University of Science and Technology, Vietnam Academy of Science and Technology, 18 Hoang Quoc Viet Road, Cau Giay, Hanoi, Vietnam Institute of Ecology and Biological Resources and Graduate University of Science and Technology, Vietnam Academy of Science and Technology, 18 Hoang Quoc Viet Road, Cau Giay Hanoi Vietnam; 3 Vu Quang National Park, Vu Quang Town, Vu Quang Distrct, Ha Tinh Province, Ha Tinh, Vietnam Vu Quang National Park, Vu Quang Town, Vu Quang Distrct, Ha Tinh Province Ha Tinh Vietnam; 4 Faculty of Environmental Sciences, University of Science, Vietnam National University, Hanoi, 334 Nguyen Trai Road, Hanoi City, Vietnam Faculty of Environmental Sciences, University of Science, Vietnam National University, Hanoi, 334 Nguyen Trai Road Hanoi City Vietnam

**Keywords:** distribution, frogs, morphology, taxonomy, Vu Quang National Park

## Abstract

**Background:**

Since the establishment of the Vu Quang National Park in 2002 in Ha Tinh Province, central Vietnam, several surveys on the amphibian fauna have been undertaken in this protected area. In 2009, Nguyen et al. provided a list of 23 amphibian species from Vu Quang National Park. In addition, a new species was described in 2021 from the National Park, namely *Vietnamophrynevuquangensis*.

**New information:**

As a result of our field surveys in 2013 and 2023, a total of 29 species of amphibians was recorded from the Vu Quang National Park. Six of them are recorded for the first time from Ha Tinh Province, comprising three species of Megophryidae, one species of Dicroglossidae, one species of Ranidae and one species of Rhacophoridae. In addition, we provide morphological data and ecological notes on the aforementioned species.

## Introduction

The Vu Quang National Park (NP), located in Ha Tinh Province of Vietnam, was established by Decision No. 102/TTg of the Prime Minister on 30 July 2002 with an area of 55,029 hectares (Tordoff et al. 2004). The Park was recognised as an ASEAN Heritage Park in October 2019. Vu Quang NP is situated in the northern part of the Truong Son Range at elevations between 100 and 2,200 m.

In terms of amphibian diversity, Ha Tinh Province is one of the most poorly-studied provinces in Vietnam. In their herpetofaunal list of Vietnam, Nguyen et al. (2009) recorded 26 species of amphibians from Ha Tinh Province and most of them were reported from Vu Quang NP and Ho Ke Go Nature Reserve (Suppl. material 1). Further new records of amphibians from Ha Tinh Province were published by Dau et al. (2015), Thai (2017), Ziegler et al. (2022) and Sheridan et al. (2023) (Suppl. material 1). Most recently, a new species was described from Vu Quang NP, viz. *Vietnamophrynevuquangensis* Hoang, Jiang, Nguyen, Orlov, Le, Nguyen, Nguyen, Nguyen, Nguyen and Ziegler (Hoang et al. 2021).

Based on our recent fieldwork in Vu Quang NP, Ha Tinh Province, Vietnam in 2013 and 2023, we herein report six new provincial records of amphibians for Ha Tinh Province.

## Materials and methods

**Sampling**

**Figure 1.**
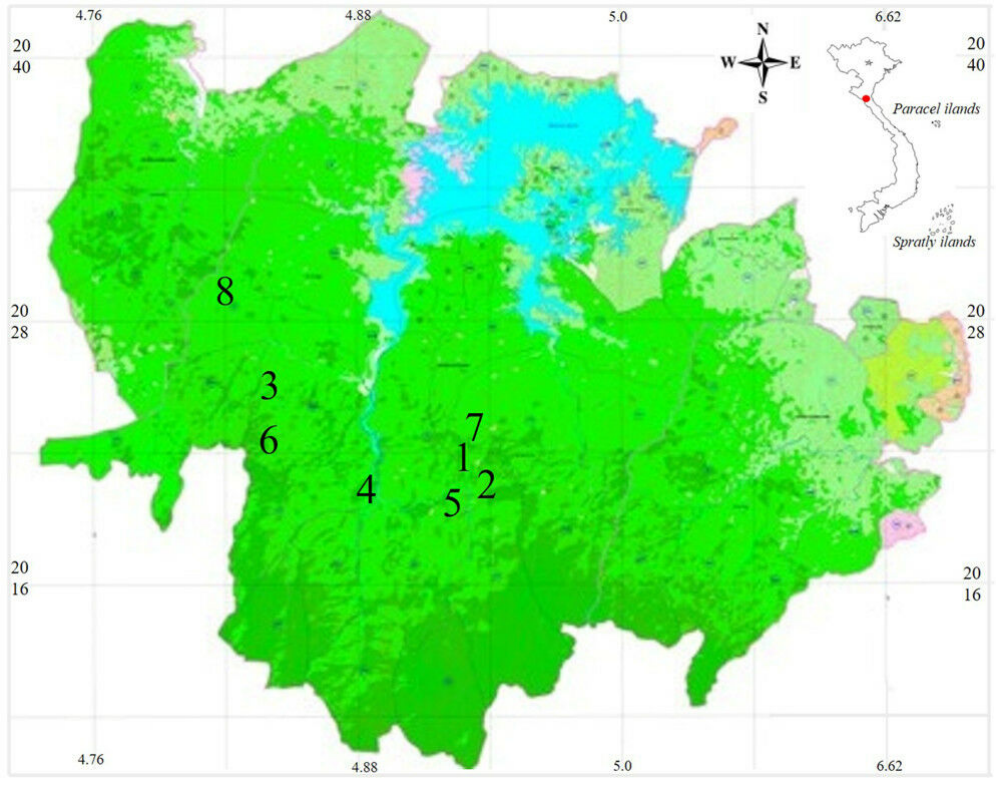
New distribution records of amphibians in Vu Quang National Park, Ha Tinh Province, Vietnam: 1 = *Leptobrachiummasatakasatoi*; 2 = Xenophryscf.parva; 3 = *Xenophryslancangica*; 4 = Quasipaacf.verrucospinosa; 5, 6 = *Amolopscompotrix*; 7, 8 = *Kurixalusodontotarsus*.

Field surveys were conducted in Vu Quang NP, Ha Tinh Province, Vietnam from 15 to 30 June 2013 by Dau QV, Thai CT, Nguyen VH and Tran DA and from 18 to 27 December 2023 by Pham VA and Tran DA The coordinates (WGS 84) and elevations were determined by using the GPS Garmin 62SX (Fig. 1).

Specimens were collected by hand between 19:00 and 22:00 h pm. After taking live photographs, specimens were euthanised in a closed vessel with a piece of cotton wool containing ethyl acetate (Simmons 2002), fixed in 80% ethanol for five hours and then transferred to 70% ethanol for permanent storage. Voucher specimens were subsequently deposited in the collections of the Faculty of Natural Sciences, Hong Duc University (HDU), Thanh Hoa Province; Faculty of Environmental Sciences, University of Science, Vietnam National University, Hanoi (VNU) and Vu Quang National Park (VQNP), Ha Tinh Province, Vietnam.

**Morphological characters**

Measurements were taken with a digital caliper to the nearest 0.1 mm. Abbreviations are as follows: (SVL) snout-vent length; (HL) head length, from posterior corner of mandible to tip of snout; (HW) maximum head width, at the angle of jaws; (RL) rostral length, from anterior corner of orbit to tip of snout; (IN) internarial distance; NS: distance from anterior edge of nostril to tip of snout; (EN) distance from anterior corner of eye to posterior edge of nostril; (ED) eye length, from anterior corner to posterior corner of eye; (IOD) minimum distance between upper eyelids; (UEW) maximum width of upper eyelid; (TD) maximum tympanum diameter; (TYE) tympanum-eye distance, from anterior margin of tympanum to posterior corner of the eye); (HAL) hand length, from elbow to tip of third finger; (FL) thigh length, from vent to knee; (TbL) shank length. For webbing formula, we followed Glaw and Vences (2007). Sex was determined by gonadal inspection in these frogs are all adults. (JJLR) Jodi J. L. Rowley; DQV (Dau Quang Vinh); HT (Ha Tinh).

## Taxon treatments

### Leptobrachium masatakasatoi

Matsui, 2013

316E51E4-CA2F-57A2-9848-A30EB9330CED

#### Materials

- **Type status:** Other material. **Occurrence:** catalogNumber: HDU 03111; individualCount: 1; sex: male; lifeStage: adult; occurrenceID: 2CE20B14-6A4E-5F9F-99AF-C88DC30BA948; **Taxon:** scientificNameID: *Leptobrachiummasatakasatoi*; scientificName: *Leptobrachiummasatakasatoi*; class: Amphibia; order: Anura; family: Megophryidae ; genus: Leptobrachium; specificEpithet: *masatakasatoi*; scientificNameAuthorship: Matsui, 2013; **Location:** country: Vietnam; countryCode: VN; stateProvince: Ha Tinh; county: Ha Tinh; municipality: Vu Quang; locality: near Huong Quang Commune; verbatimElevation: 1294 m; verbatimLatitude: 18°14'13.67"N; verbatimLongitude: 105°25'54.23"E; verbatimCoordinateSystem: WGS84; **Event:** eventDate: Jume; eventTime: 2013; eventRemarks: collected by V. Q. Dau and T. C. Thai; **Record Level:** language: en; collectionCode: Amphibians; basisOfRecord: PreservedSpecimen

#### Description

Morphological characters of the specimen from Ha Tinh Province matched descriptions of Matsui (2013) and Pham et al. (2016): Body elongate, SVL 58.6 mm in male (n = 1). Head wider than long (HL 25.6 mm, HW 26.1 mm); snout round, barely projecting beyond upper jaw (RL 10.5 mm), longer than horizontal diameter of eye (ED 8.2 mm); canthus sharp, straight; loreal region oblique, moderately concave; nostril lateral, closer to tip of snout than to eye (NS 4.8 mm, EN 5.7 mm); interorbital space flat, broader than upper eyelid and internarial distance (IOD 7.1 mm, UEW 6.4 mm, IN 4.4 mm); tympanum indistinct; vomerine teeth absent; tongue heart-shaped, notched posteriorly; vocal openings absent. Fore-limb long (HAL 33.7 mm); relative finger lengths II < IV < I < III; fingers without dermal fringe, free of webbing; tips of fingers round, slightly swollen; palmar tubercles two, oval; nuptial pads absent. Hind-limb slender, thigh longer than tibia (FL 26.7 mm, TbL 22.6 mm, TbL/SVL 38.5%); relative toe lengths I < II < V< III < IV; tips of toes slightly swollen; webbing formula I1–1II1–1III1–2IV2–1V; tibiotarsal articulation reaching to posterior margin of tympanum when limb adpressed along body; inner metatarsal tubercle distinct, as long as of toe I.

Skin: Dorsal surface with fine network of ridges, tubercles present in the posterior region of sacrum, more distinct in anterior part of vent; upper lip without spines in male; supratympanic fold present, from posterior edge of eye to axilla; flanks granular; throat and chest asperities; belly and ventral surfaces of limbs smooth, except for granular thigh.

**Figure 2.**
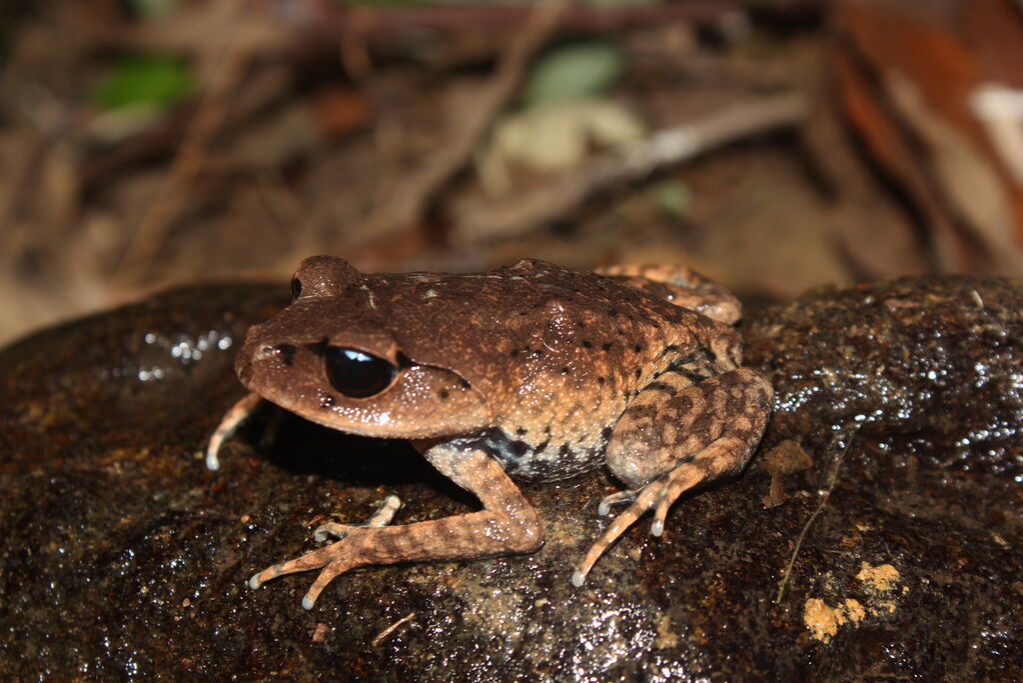
*Leptobrachiummasatakasatoi* (adult male) from Ha Tinh Province, Vietnam. Photo by V. Q. Dau.

Colouration in life: Dorsal surface of head reddish-brown, with some dark spots on upper eyelid, back with irregularly black spots; supratympanic fold edged in black below; flank light brown with large black spots; anterior part of thigh with large dark spots; dorsal limb light brown with dark crossbars; ventral surface with irregular brown and cream markings (Fig. 2).

#### Distribution

In Vietnam, this species was recorded from Son La and Nghe An Provinces (Pham et al. 2016, Do et al. 2017). Elsewhere, this species is known from Laos (Frost 2024).

#### Ecology

The specimen was found on the ground near a small stream at 19:15 h pm. The surrounding habitat was evergreen forest of large hardwood and shrubs.

### Xenophrys parva

(Boulenger, 1893)

731B7838-6DCD-5E10-BF7A-178FF7CA316D

#### Materials

- **Type status:** Other material. **Occurrence:** catalogNumber: JJLR 00043; individualCount: 1; sex: male; lifeStage: adult; occurrenceID: BF6DE9C4-2C11-5A70-A930-B1BAF9009DB3; **Taxon:** scientificNameID: Xenophryscf.parva; scientificName: Xenophryscf.parva; class: Amphibia; order: Anura; family: Megophryidae; genus: Xenophrys; specificEpithet: parva; scientificNameAuthorship: (Boulenger, 1893); **Location:** country: Vietnam; countryCode: VN; stateProvince: Ha Tinh; county: Ha Tinh; municipality: Vu Quang; locality: near Huong Quang Commune; verbatimElevation: 1214 m; verbatimLatitude: 18°14'36.82"N; verbatimLongitude: 105°25'56.42"E; verbatimCoordinateSystem: WGS84; **Event:** eventDate: June; eventTime: 2013; eventRemarks: collected by V. Q. Dau and T. C. Thai; **Record Level:** language: en; collectionCode: Amphibians; basisOfRecord: PreservedSpecimen

#### Description

Morphological characters of the specimen from Ha Tinh Province relatively matched descriptions of Boulenger (1893), Bourret (1942), Fei et al. (2009), Pham et al. (2019a) and Lyu et al. (2023): SVL 41.8 mm in male (n = 1); head longer than wide (HL 15.4, HW 15.0 mm, HL/SVL 36.8%, HW/SVL 35.9%); snout obliquely truncate, projecting beyond upper jaw (RL 5.5 mm), longer than horizontal diameter of eye (ED 4.8 mm); loreal region concave; canthus rostralis well developed; nostril lateral, slightly closer to eye than to tip of snout (NS 2.8 mm, EN 2.5 mm); interorbital space flat, broader than upper eyelid and narrower than internarial distance (IOD 4.3 mm, UEW 3.9 mm, IN 4.8 mm); tympanum distinct (TD 3.0 mm); vomerine teeth present; tongue heart-shaped, round posteriorly; vocal in male present.

Fore-limb slender (HAL 15.7 mm); relative finger lengths I ≤ II < IV < III; fingers without dermal fringe, free of webbing; tips of fingers round, not swollen; subarticular tubercles indistinct; palmar tubercles indistinct; nuptial pads absent in males. Hind-limb slender, tibia longer than thigh (TbL 19.8 mm, FL 18.5 mm); relative toe lengths I < II < V < III < IV; tips of toes round, slightly swollen; webbing rudiment; toes dermal fringe absent; metatarsal tubercle indistinct; subarticular tubercles indistinct; tibiotarsal articulation reaching to anterior corner of eye when limb adpressed along body.

Skin: Dorsal surface smooth, with sparse small granules; dorsum with a weak discontinuous V-shaped ridge; dorsolateral ridges discontinuous; flanks with small glandular warts; supratympanic fold present, from posterior corner of eye to axilla; outer margin of upper eyelid with weak medial bumped appendage; around cloaca with small tubercles; ventral surface smooth.

**Figure 3.**
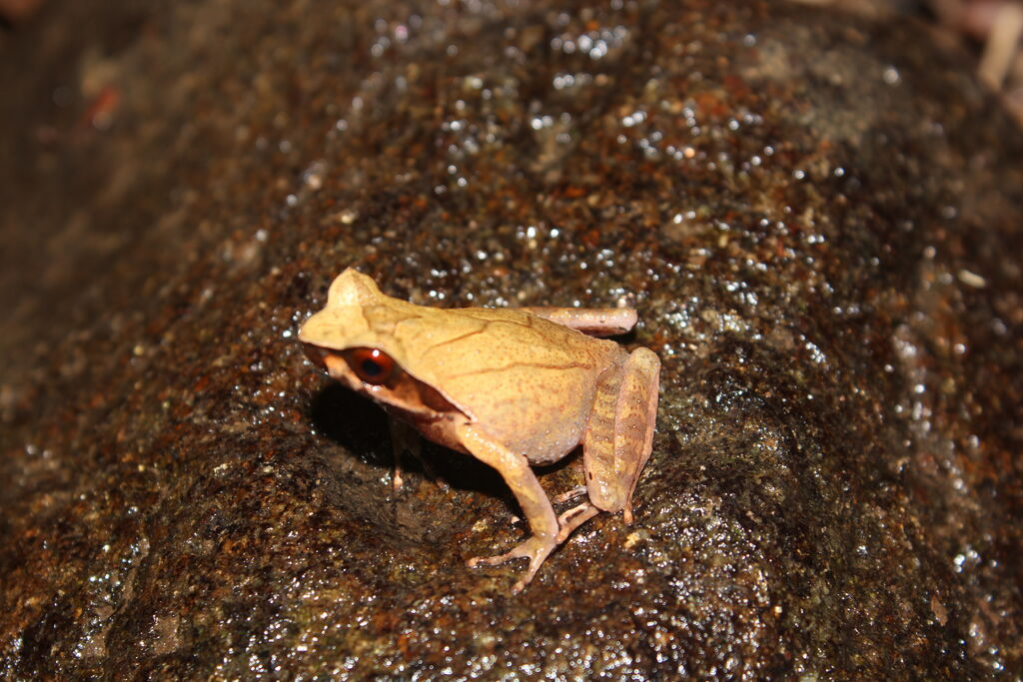
Xenophryscf.parva (adult male) from Ha Tinh Province, Vietnam. Photo by V. Q. Dau.

Colouration in life: Dorsal surface light yellowish-brown; a dark brown triangular marking between eyes; upper lip with vertical dark bars; ventral surface white, a round white spot present on each side of the chest; ventral surface of limbs reddish (Fig. 3).

#### Distribution

In Vietnam, this species has been recorded from Dien Bien, Lai Chau, Lao Cai, Ha Giang, Son La and Thanh Hoa Provinces (Nguyen et al. 2009, Orlov et al. 2015, Luong et al. 2019, Pham et al. 2019a and Frost 2024). Elsewhere, this species is known from China, Myanmar, Laos and Thailand (Lyu et al. 2023) (see discussion).

#### Ecology

The specimen was found on a tree near a small stream at 22:00 h pm. The surrounding habitat was evergreen forest of medium hardwoods and shrubs.

#### Notes

The specimen from Ha Tinh differs from the description of Lyu et al. (2023) in having relative finger lengths I ≤ II < IV < III (vs. II < IV < I < III) and dorsum with a small dark X-shaped marking (vs. larger dark X-shaped marking).

### Xenophrys lancangica

Lyu, Wang & Wang, 2023

232D458C-23A7-5655-9C84-06058C8A7647

#### Materials

- **Type status:** Other material. **Occurrence:** catalogNumber: DQV 0085; individualCount: 1; sex: male; lifeStage: adult; occurrenceID: D973A0ED-6F81-5A0B-A72B-717AF63D19E0; **Taxon:** scientificNameID: *Xenophryslancangica*; scientificName: *Xenophryslancangica*; genus: Xenophrys; specificEpithet: *lancangica*; scientificNameAuthorship: Lyu, Wang & Wang, 2023; **Location:** country: Vietnam; countryCode: VN; stateProvince: Ha Tinh; county: Ha Tinh; municipality: Vu Quang; locality: near Huong Quang Commune; verbatimElevation: 260m; verbatimLatitude: 18°20'29.72"N; verbatimLongitude: 105°14'29.27"E; verbatimCoordinateSystem: WGS84; **Event:** eventDate: Jume; eventTime: 2013; eventRemarks: collected by V. Q. Dau and T. C. Thai; **Record Level:** language: en; collectionCode: Amphibians; basisOfRecord: PreservedSpecimen

#### Description

Morphological characters of the specimen from Ha Tinh Province matched the description of Lyu et al. (2023): SVL 67.6 mm in male (n = 1); head wider than long (HL 24.8, HW 25.1 mm, HL/SVL 36.7%, HW/SVL 37.1%); snout obliquely truncate, projecting beyond upper jaw (RL 10.1 mm), as long as horizontal diameter of eye (ED 10.0 mm); canthus rostralis well developed; loreal region concave; nostril lateral, closer to eye than to tip of snout (NS 5.1 mm, EN 4.7 mm); interorbital space flat, broader than upper eyelid and narrower than internarial distance (IOD 10.5 mm, UEW 7.1 mm, IN 4.6 mm); tympanum distinct (TD 5.2 mm); vomerine teeth present; tongue weakly notched posteriorly; vocal sac present in male. Fore-limb slender (HAL 33.2 mm); relative finger lengths II < IV < I < III; fingers without dermal fringe, free of webbing; tips of fingers round, slightly swollen; subarticular tubercles indistinct; palmar tubercles indistinct; nuptial pads on dorsal bases of fingers I and II. Hind-limb slender, tibia longer than thigh (TbL 37.0 mm, FL 34.6 mm); relative toe lengths I < II < V < III < IV; tips of toes round, slightly swollen; webbing rudiment; toes dermal narrow lateral fringes; metatarsal tubercle indistinct; subarticular tubercles indistinct; inner and outer metatarsal tubercles indistinct; tibiotarsal articulation reaching to the position between nostril and tip of snout when limb adpressed along body.

Skin: Dorsal surface smooth with sparse small granules; a weak discontinuous X- shaped ridge on centre of dorsum, dorsolateral ridges present; outer margin of upper eyelid with a weak medial bumped appendage; flank with sparse large tubercles; supratympanic fold present, from posterior edge of eye to axilla; ventral surface smooth.

**Figure 4.**
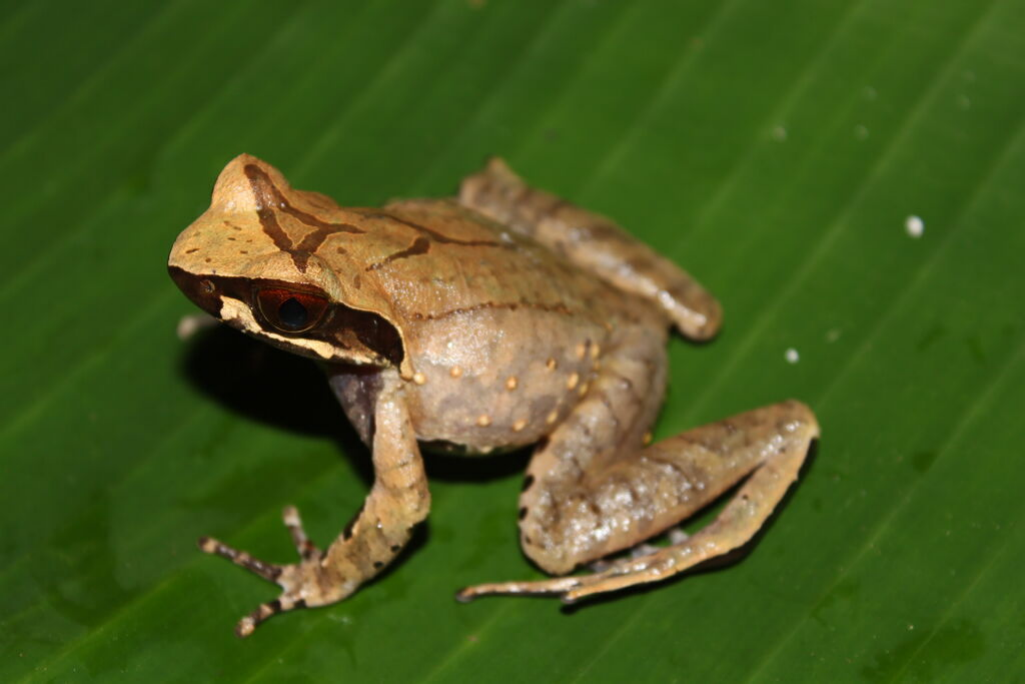
*Xenophryslancangica* (adult male) from Ha Tinh Province, Vietnam. Photo by V. Q. Dau.

Colouration in life: Dorsal surface brown-yellowish, with a triangular marking between eyes, a Y-shaped marking on centre of dorsum; dorsal limb with dark transverse bands; throat and chest brown, with a round white spot on each side of the chest present; ventral surface whitish, ventral surface of limbs reddish (Fig. 4).

#### Distribution

In Vietnam, this species is knnown from Dien Bien, Phu Tho, Thanh Hoa, Nghe An, Quang Binh and Quang Tri Provinces (Frost 2024). Elsewhere, this species is known from China, Laos and Thailand (Frost 2024).

#### Ecology

The specimen was found on the tree at 20:24 h pm. The surrounding habitat was evergreen forest of medium hardwoods and shrubs.

#### Notes

*Xenophryslancangica* closely resembles *X.maosonensis* and *X.truongsonensis*, but it differs from *X.maosonensis* by having the tibiotarsal articulation reaching to the position between nostril and tip of snout (vs. reaching to centre of eye in *X.maosonensis*) (Lyu et al. 2023) and from *X.truongsonensis* by having flank with large tubercles (vs. with small tubercles in *X.truongsonensis*); toes lateral fringes absent (vs. toes with narrow lateral fringes); and white spots on flank and back of thigh larger than those in *X.truongsonensis* (Luong et al. 2022).

### Quasipaa verrucospinosa

(Bourret, 1937)

D6838855-A1FC-54F8-995C-72DEF1B8F621

#### Materials

- **Type status:** Other material. **Occurrence:** catalogNumber: HT.2023.27; individualCount: 1; sex: female; lifeStage: adult; occurrenceID: 0A905E19-8D9C-5061-96D1-92FF26553133; **Taxon:** scientificNameID: Quasipaacf.verucospinosa; scientificName: Quasipaacf.verucospinosa; genus: Quasipaa; specificEpithet: *verucospinosa*; scientificNameAuthorship: (Bourret, 1937); **Location:** country: Vietnam; countryCode: VN; stateProvince: Ha Tinh; county: Ha Tinh; municipality: Vu Quang; locality: near Huong Quang Commune; verbatimElevation: 240 m; verbatimLatitude: 18°17'13.3"N; verbatimLongitude: 105°22'08.1"E; verbatimCoordinateSystem: WGS84; **Event:** eventDate: December; eventTime: 2023; eventRemarks: collected by A.V Pham and A. D Tran; **Record Level:** language: en; collectionCode: Amphibians; basisOfRecord: PreservedSpecimen
- **Type status:** Other material. **Occurrence:** catalogNumber: HT.2023.28; individualCount: 1; sex: female; lifeStage: adult; occurrenceID: 50A86991-6867-504B-9D0B-7A5F02CBE588; **Taxon:** scientificNameID: Quasipaacf.verucospinosa; scientificName: Quasipaacf.verucospinosa; genus: Quasipaa; specificEpithet: *verucospinosa*; scientificNameAuthorship: (Bourret, 1937); **Location:** country: Vietnam; countryCode: VN; stateProvince: Ha Tinh; county: Ha Tinh; municipality: Vu Quang; locality: near Huong Quang Commune; verbatimElevation: 240 m; verbatimLatitude: 18°17'13.3"N; verbatimLongitude: 105°22'08.1"E; verbatimCoordinateSystem: WGS85; **Event:** eventDate: January; eventTime: 2024; eventRemarks: collected by A.V Pham and A. D Tran; **Record Level:** language: en; collectionCode: Amphibians; basisOfRecord: PreservedSpecimen
- **Type status:** Other material. **Occurrence:** catalogNumber: HT.2023.29; individualCount: 1; sex: female; lifeStage: adult; occurrenceID: E09D4017-003D-50E5-B90B-98C4FFB9CDE5; **Taxon:** scientificNameID: Quasipaacf.verucospinosa; scientificName: Quasipaacf.verucospinosa; genus: Quasipaa; specificEpithet: *verucospinosa*; scientificNameAuthorship: (Bourret, 1937); **Location:** country: Vietnam; countryCode: VN; stateProvince: Ha Tinh; county: Ha Tinh; municipality: Vu Quang; locality: near Huong Quang Commune; verbatimElevation: 240 m; verbatimLatitude: 18°17'13.3"N; verbatimLongitude: 105°22'08.1"E; verbatimCoordinateSystem: WGS86; **Event:** eventDate: February; eventTime: 2025; eventRemarks: collected by A.V Pham and A. D Tran; **Record Level:** language: en; collectionCode: Amphibians; basisOfRecord: PreservedSpecimen

#### Description

Morphological characters of the specimens from Ha Tinh Province matched the descriptions of Bourret (1937), Bourret (1942) and Fei et al. (2009): Body large, SVL 76.0–95.9 mm in females (n = 3). Head wider than long (HL 30.3–37.0 mm, HW 31.9–39.9 mm; HL/SVL 38.6–40.5%; HW/SVL 41.6–43.5%); snout round, barely projecting beyond upper jaw (RL 12.2–15.0 mm), longer than horizontal diameter of eye (ED 10.6–12.9 mm); canthus rostralis indistinct; loreal region oblique, concave; nostril lateral, closer to eye than to tip of snout (NS 7.2–8.4 mm, EN 5.8–6.4 mm); interorbital space flat, narrower than upper eyelid and internarial distance (IOD 6.3–7.8 mm, UEW 7.7–69.0 mm, IN 8.0–8.8 mm); tympanum visible (TD 3.0–3.3 mm), smaller than the distance from tympanum to eye (TYE 4.7–6.5 mm), about 26–28% eye diameter; vomerine teeth present; tongue cordiform, notched posteriorly. Fore-limb short (HAL 31.2–41.8 mm); relative finger lengths II < I < IV < III; fingers free of webbing; fingers II and III with narrow lateral dermal fringes; tips of fingers round, slightly swollen; palmar tubercles two, inner metatarsal tubercle oval, outer metatarsal tubercle elongate. Hind-limb: thigh shorter than tibia (FL 39.5–50.5 mm, TbL 45.0–54.5 mm); relative toe lengths I < II < V < III < IV; tips of toes swollen; toes fully webbed; outer sides of toes I and V with dermal fringe; tibiotarsal articulation reaching to nostril when limb adpressed along body; inner metatarsal tubercle elongate, outer metatarsal tubercle absent.

Skin: Dorsal surface with oval, round and elongate tubercles; flank covered by oval and round tubercles; supratympanic fold present, from posterior corner of eye to angle of jaw; ventral surface smooth.

**Figure 5.**
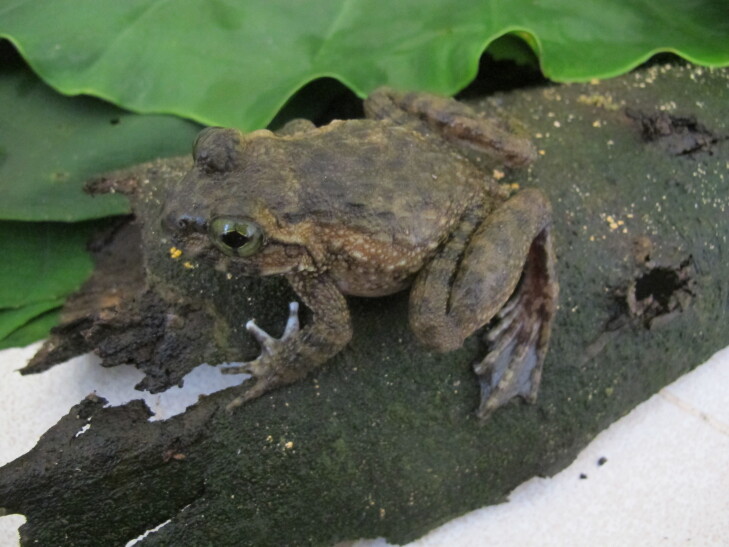
Quasipaacf.verrucospinosa (adult female) from Ha Tinh Province, Vietnam. Photo by A. V. Pham.

Colouration in life: Dorsal surface yellowish-brown; dorsal limbs light brown with indistinct dark crossbars; throat white with brown markings; ventral surface of limbs, chest and belly immaculate white (Fig. 5).

#### Distribution

In Vietnam, this species was recorded from Dien Bien, Lao Cai, Son La, Vinh Phuc, Nghe An, Thua Thien Hue and Kon Tum Provinces (Frost 2024). Elsewhere, this species is known from Laos (Frost 2024).

#### Ecology

All specimens were found near waterfalls in rocky streams between 20:00 and 22:00 h pm. The surrounding habitat was evergreen forest of large and medium hardwoods mixed with shurbs.

#### Notes

Our specimens from Ha Tinh Province slightly differ from those in descriptions of Bourret (1942) and Inger et al. (1999) by having inner metatarsal tubercle oval (vs. inner metatarsal tubercle round); different dorsal pattern (yellowish-brown vs. yellowish-grey); different ventral colour pattern (immaculate white vs. light yellow); and iris dark green (vs. pale copper).

### Amolops compotrix

(Bain, Stuart & Orlov, 2006)

5686ED7F-A605-5C93-BD47-9DA16CAE5184

#### Materials

- **Type status:** Other material. **Occurrence:** catalogNumber: HDU 03109; individualCount: 1; sex: female; lifeStage: adult; occurrenceID: CB80C67B-FEA6-560C-81C2-E7ECB9D4B9A2; **Taxon:** scientificNameID: *Amolopscompotrix*; scientificName: *Amolopscompotrix*; class: Amphibia; order: Anura; family: Ranidae; genus: Amolops; specificEpithet: *compotrix*; scientificNameAuthorship: (Bain, Stuart, and Orlov, 2006); **Location:** country: Vietnam; countryCode: VN; stateProvince: Ha Tinh; county: Ha Tinh; municipality: Vu Quang; locality: near Huong Quang Commune; verbatimElevation: 1330 m; verbatimLatitude: 18°14'10.68"N; verbatimLongitude: 105°25'54.05"E; verbatimCoordinateSystem: WGS84; **Event:** eventDate: Jume; eventTime: 2013; eventRemarks: collected by V. Q. Dau and T. C. Thai; **Record Level:** language: en; collectionCode: Amphibians; basisOfRecord: PreservedSpecimen
- **Type status:** Other material. **Occurrence:** catalogNumber: HDU 03110; individualCount: 1; sex: male; lifeStage: adult; occurrenceID: F3F59600-071E-5026-8033-B9C07BEF908F; **Taxon:** scientificNameID: *Amolopscompotrix*; scientificName: *Amolopscompotrix*; class: Amphibia; order: Anura; family: Ranidae; genus: Amolops; specificEpithet: *compotrix*; scientificNameAuthorship: (Bain, Stuart, and Orlov, 2006); **Location:** country: Vietnam; countryCode: VN; stateProvince: Ha Tinh; county: Ha Tinh; municipality: Vu Quang; locality: near Huong Quang Commune; verbatimElevation: 1330 m; verbatimLatitude: 18°14'10.68"N; verbatimLongitude: 105°25'54.05"E; verbatimCoordinateSystem: WGS84; **Event:** eventDate: Jume; eventTime: 2013; eventRemarks: collected by V. Q. Dau and T. C. Thai; **Record Level:** language: en; collectionCode: Amphibians; basisOfRecord: PreservedSpecimen
- **Type status:** Other material. **Occurrence:** catalogNumber: HDU 03149; individualCount: 1; sex: male; lifeStage: adult; occurrenceID: 3A2A6808-B947-5573-8445-D79FA028D20C; **Taxon:** scientificNameID: *Amolopscompotrix*; scientificName: *Amolopscompotrix*; class: Amphibia; order: Anura; family: Ranidae; genus: Amolops; specificEpithet: *compotrix*; scientificNameAuthorship: (Bain, Stuart, and Orlov, 2006); **Location:** country: Vietnam; countryCode: VN; stateProvince: Ha Tinh; county: Ha Tinh; municipality: Vu Quang; locality: near Huong Quang Commune; verbatimElevation: 1021 m; verbatimLatitude: 18°17'41.03"N; verbatimLongitude: 105°17'34.30"E; verbatimCoordinateSystem: WGS84; **Event:** eventDate: Jume; eventTime: 2013; eventRemarks: collected by V. Q. Dau and T. C. Thai; **Record Level:** language: en; collectionCode: Amphibians; basisOfRecord: PreservedSpecimen

#### Description

Morphological characters of the specimens from Ha Tinh Province matched the description of Bain et al. (2006): Body elongate, SVL 40.6–42.0 mm in males (n = 2) and 60.2 mm in the female (n = 1). Head longer than wide (HL 15.4–17.3 mm, HW 13.4–15.0 mm in males; HL 22.0 mm, HW 18.6 mm in the female); snout obtusely pointed in dorsal view, projecting beyond lower jaw (RL 6.3–6.8 mm in males and 9.4 mm in the female), longer than horizontal diameter of eye (ED 6.0–6.5 mm in males and 8.4 mm in the female); canthus rostralis distinct, slightly constricted behind nostrils; lores concave and oblique; nostril lateral, midway between tip of snout and eye (NS 2.7–2.8 mm, EN 2.8–3.0 mm in males and NS 4.3 mm, EN 4.1 mm in the female); interorbital distance narrower than upper eyelid and internarial distance (IOD 3.7–3.8 mm, UEW 4.6–4.8 mm, IN 3.9–4.4 mm in males and IOD 4.5 mm, UEW 5.8 mm, IN 5.2 mm in the female); pineal body distinct; tympanum distinct, round, 43% of eye diameter in males or 40% of eye diameter in the female; vomerine teeth present; tongue cordiform, deeply notched posteriorly; vocal openings on floor of mouth at corner. Fore-limb long (HAL 21.3–23.7 mm in males, 30.8 mm in the female); relative finger lengths I < II < IV < III; fingers without dermal fringe, free of webbing; tips of fingers expanded with circum-marginal grooves; palmar tubercles two, oval, in contact; nuptial pads present. Hind-limb slender, thigh shorter than tibia (FL 24.3–25.3 mm, TbL 24.7–27.4 mm in males and FL 35.5 mm, TbL 37.9 in the female); relative toe lengths I < II < V < III < IV; tips of toes expanded, width of toe IV disc equal to that of finger III disc; webbing formula I0–0II0–0III0–1IV1–0V; tibiotarsal articulation reaching to beyond tip of snout when limb adpressed along body; inner metatarsal tubercle distinct, elongate, oval; outer metatarsal tubercle round, small.

Skin: Dorsal surface smooth; supratympanic fold present, from posterior edge of eye to axilla; glandular dorsolateral fold present; flank smooth; ventral surface smooth.

**Figure 6.**
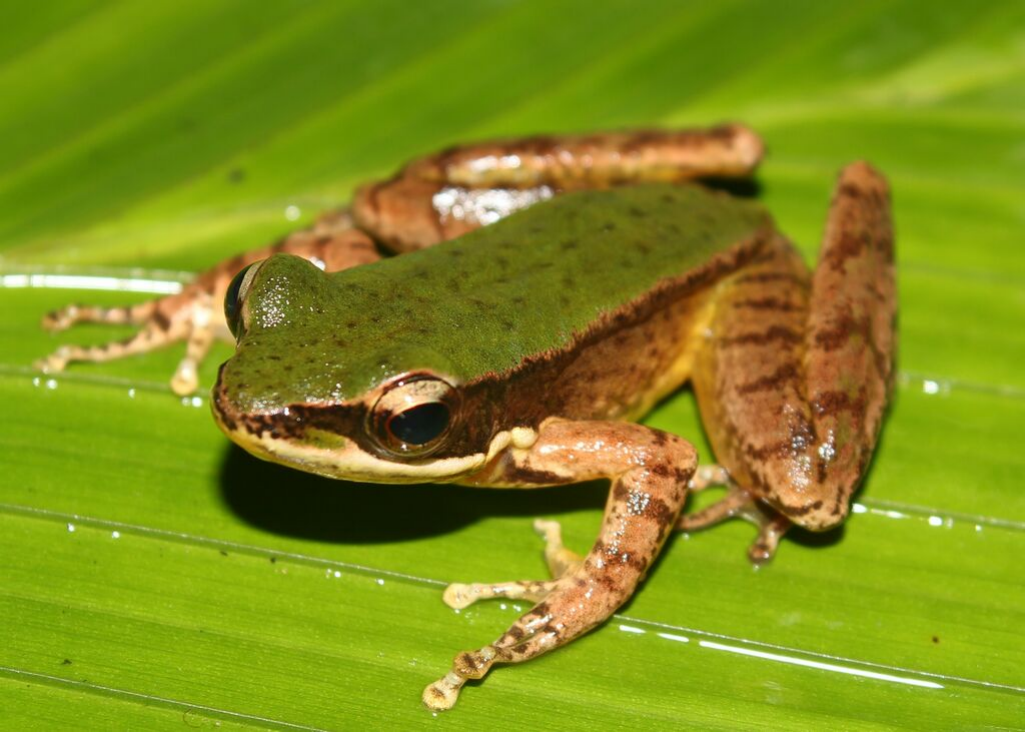
*Amolopscompotrix* (adult male) from Ha Tinh Province, Vietnam. Photo by V. Q. Dau.

Colouration in life: Dorsal surface green, with dark brown speckling diurnally, lateral side of body dark brown below edge of dorsolateral fold; lip stripe white, from tip of snout to posterior of arm insertion; dorsal surface of limbs greyish-brown with dark crossbars; ventral surface of jaw, throat, chest and belly white; ventral surface of limbs cream (Fig. 6).

#### Distribution

In Vietnam, this species was recorded from Nghe An, Quang Binh, Thua Tien Hue and Kon Tum Provinces (Pham et al. 2019b, Dau et al. 2020 and Frost 2024). Elsewhere, this species is known from Laos (Frost 2024).

#### Ecology

All specimens were found on a leaf or tree branches near small streams between 19:30 and 21:00 h pm. The surrounding habitat was evergreen forest of large hardwoods and shrubs.

### Kurixalus odontotarsus

(Ye & Fei, 1993)

52A9DE7A-8BFD-5200-88E3-599F71E64109

#### Materials

- **Type status:** Other material. **Occurrence:** catalogNumber: HDU 03120; individualCount: 1; sex: male; lifeStage: adult; occurrenceID: DFCD61C5-110D-5E34-AC35-E6109A63B719; **Taxon:** scientificNameID: *Kurixalusodontotarsus*; scientificName: *Kurixalusodontotarsus*; class: Amphibia; order: Anura; family: Rhacophoridae; genus: Kurixalus; specificEpithet: *odontotarsus*; scientificNameAuthorship: Nguyen, Duong, Luu, and Poyarkov, 2020; **Location:** country: Vietnam; countryCode: VN; stateProvince: Ha Tinh; county: Ha Tinh; municipality: Vu Quang; locality: near Huong Quang Commune; verbatimElevation: 631 m; verbatimLatitude: 18°15'41.09"N; verbatimLongitude: 105°26'38.14"E; verbatimCoordinateSystem: WGS84; **Event:** eventDate: Jume; eventTime: 2013; eventRemarks: collected by V. Q. Dau and T. C. Thai; **Record Level:** language: en; collectionCode: Amphibians; basisOfRecord: PreservedSpecimen
- **Type status:** Other material. **Occurrence:** catalogNumber: HDU 03144; individualCount: 1; sex: female; lifeStage: adult; occurrenceID: 57636F02-A6E9-505F-99EF-9F9495AD607C; **Taxon:** scientificNameID: *Kurixalusodontotarsus*; scientificName: *Kurixalusodontotarsus*; class: Amphibia; order: Anura; family: Rhacophoridae; genus: Kurixalus; specificEpithet: *odontotarsus*; scientificNameAuthorship: Nguyen, Duong, Luu, and Poyarkov, 2020; **Location:** country: Vietnam; countryCode: VN; stateProvince: Ha Tinh; county: Ha Tinh; municipality: Vu Quang; locality: near Huong Quang Commune; verbatimElevation: 290 m; verbatimLatitude: 18°20'43.04"N; verbatimLongitude: 105°17'46.03"E; verbatimCoordinateSystem: WGS84; **Event:** eventDate: Jume; eventTime: 2013; eventRemarks: collected by V. Q. Dau and T. C. Thai; **Record Level:** language: en; collectionCode: Amphibians; basisOfRecord: PreservedSpecimen
- **Type status:** Other material. **Occurrence:** catalogNumber: HDU 03145; individualCount: 1; sex: male; lifeStage: adult; occurrenceID: 5E9B7883-0BE4-55E4-8F5A-CDD022177708; **Taxon:** scientificNameID: *Kurixalusodontotarsus*; scientificName: *Kurixalusodontotarsus*; class: Amphibia; order: Anura; family: Rhacophoridae; genus: Kurixalus; specificEpithet: *odontotarsus*; scientificNameAuthorship: Nguyen, Duong, Luu, and Poyarkov, 2020; **Location:** country: Vietnam; countryCode: VN; stateProvince: Ha Tinh; county: Ha Tinh; municipality: Vu Quang; locality: near Huong Quang Commune; verbatimElevation: 290 m; verbatimLatitude: 18°20'43.04"N; verbatimLongitude: 105°17'46.03"E; verbatimCoordinateSystem: WGS84; **Event:** eventDate: Jume; eventTime: 2013; eventRemarks: collected by V. Q. Dau and T. C. Thai; **Record Level:** language: en; collectionCode: Amphibians; basisOfRecord: PreservedSpecimen

#### Description

Morphological characters of specimens from Ha Tinh Province matched the descriptions of Fei et al. (2009) and Wu et al. (2022): SVL 28.7–33.2 mm in males (n = 2) and 47.1 mm in the female (n = 1). Head wider than long (HL 10.8–11.4 mm, HW 11.0–12.0 mm, HL/SVL 34.3–37.5%, HW/SVL 36.0–38.2% in males; HL 17.1 mm, HW 17.4 mm, HL/SVL 36.2%, HW/SVL 37.0% in the female); snout slightly pointed, projecting beyond upper jaw (RL 4.7–5.3 mm in males and 6.7 mm in the female), longer than horizontal diameter of eye (ED 4.0–4.1 mm in males and 4.9 mm in the female); canthus blunt; loreal region oblique, concave; nostril lateral, closer to eye than to tip of snout (NS 1.7–2.2 mm, EN 2.1–2.5 mm in males and NS 2.2 mm, EN 3.5 mm in the female); interorbital space flat, broader than upper eyelid and internarial distance (IOD 3.2–3.7 mm, UEW 3.0–3.5 mm, IN 2.4–2.8 mm in males and IOD 4.4 mm, UEW 4.0 mm, IN 3.6 mm in the female); tympanum distinct, round (TD 2.0–2.3 mm in males, 3.2 mm in the female); vomerine teeth present; tongue heart-shaped, notched posteriorly; vocal single internal. Fore-limb long (HAL 14.2–16.1 mm in males, 23.3 mm in the female); relative finger lengths I < II < IV < III; fingers with lateral dermal fringes, with basal webbing; tips of finger expanded into wide discs with circum-marginal and transverse ventral grooves; palmar tubercles two; finger I of two males with nuptial pads. Hind-limb slender, thigh shorter than tibia (FL 15.1–16.5 mm, TbL 15.6–16.7 mm in males and FL 22.5 mm, TbL 22.6 in the female); relative toe lengths I < II < V < III < IV; tips of toes expanded into discs, webbing formula I2–2½II1½–3III1½–3½IV3–1½V; inner metatarsal tubercle distinct, outer metatarsal tubercle absent; tibiotarsal articulation reaching to the position between the eye and snout when limb adpressed along body.

Skin: Dorsal surface with small tubercles; supratympanic fold present, from posterior edge of eye to axilla; along outer edge of forearm with a row of warts, forming a serrated fringe; along outer edge of tarsus and metatarsus with a series of tubercles forming serrated dermal fringe; below vent with a patch of white pustules; ventral surfaces granulated.

**Figure 7.**
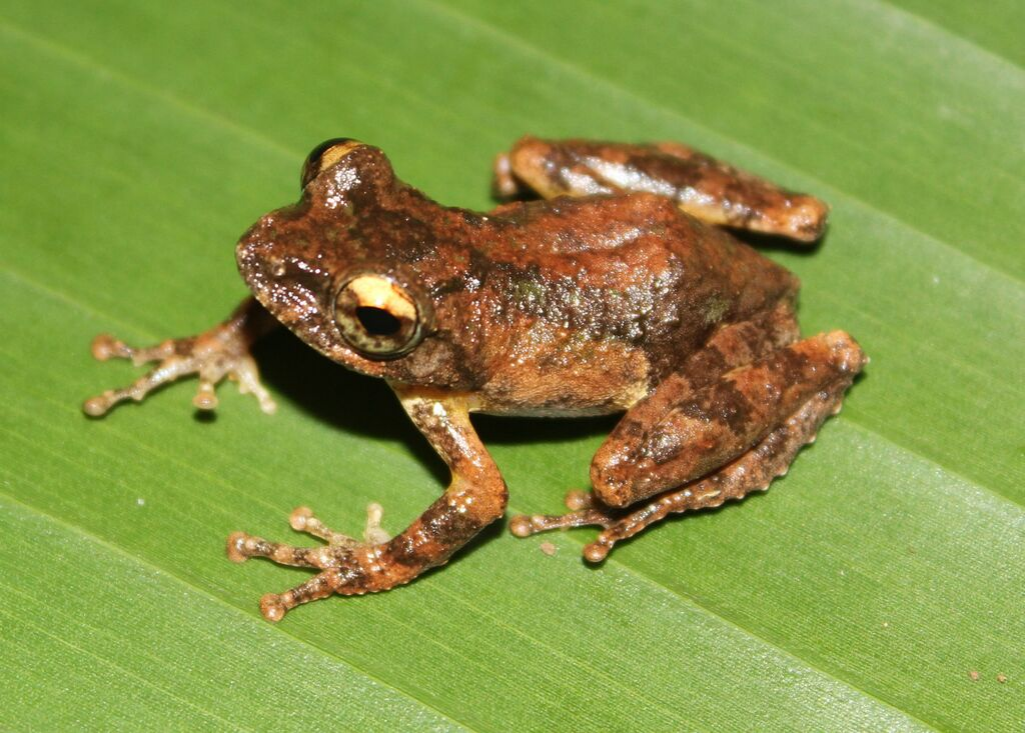
*Kurixalusodontotarsus* (adult male) from Ha Tinh Province, Vietnam. Photo by V. Q. Dau.

**Figure 8.**
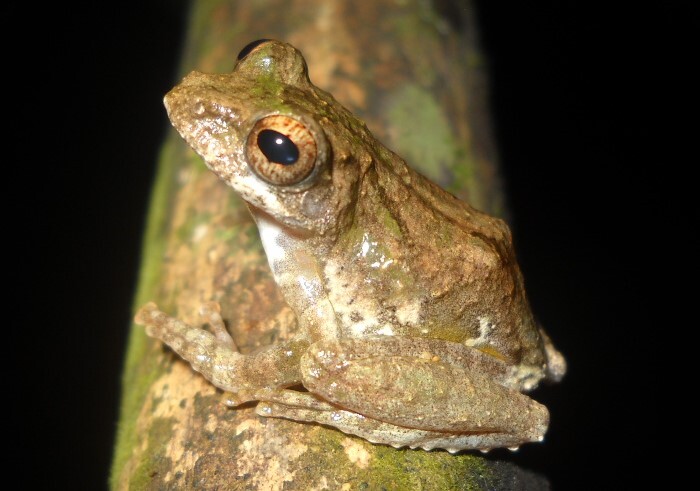
*Kurixalusodontotarsus* (adult female) from Ha Tinh Province, Vietnam. Photo by V. Q. Dau.

Colouration in life: Dorsal surface yellowish-brown or greenish-brown, with a dark-brown interorbital bar in triangle shape and a dark-brown Y-shaped saddle-like marking on the back; flank light brown with indistinct brownish spots; dorsal limbs brown with dark crossbars; a white conical projection on tibiotarsal articulation; ventral surface with dark-grey mottling, becoming denser on throat, more sparse on chest and belly (Figs 7, 8).

#### Distribution

In Vietnam, this species was recorded from Lao Cai and Ha Giang Provinces (Frost 2024). Elsewhere, this species is known from China, Laos and Thailand (Frost 2024).

#### Ecology

All specimens were found on leaves near large streams between 21:00 and 22:00 h. The surrounding habitat was evergreen forest of large hardwoods and shrubs.

## Discussion

Our new records bring the total number of amphibian species from Ha Tinh Province to 42, comprising one species of Ichthyophiidae, two species of Bufonidae, seven species of Megophryidae, six species of Microhylidae, seven species of Dicroglossidae, ten species of Ranidae and nine species of Rhacophoridae. Most of records of the amphibian fauna in Ha Tinh Province are known from Ke Go Nature Reserve (30 species) and Vu Quang NP (29 species), of which 22 species occur in both Ho Ke Go Nature Reserve and Vu Quang NP (Suppl. material 1). In terms of conservation status, Vu Quang harbours four species that are listed in the Red Data Book of Vietnam (Dang et al. 2007), with three species categorised as Endangered - EN (*Rhacophoruskio*, *Thelodermacorticale*, *Zhangixalusfeae*) and one species as Vulnerable - VU (*Ingerophrynusgaleatus*); one species (*Microhylaannamensis*) is listed as {Vulnerable} in the IUCN Red List (IUCN 2024).

According to Mahony et al. (2020), *Xenophrysparva* is only known with certainty from eastern Myanmar and the old range statement for nominal *Megophrysparva* (western to eastern Nepal and Bhutan, India through Bangladesh and Myanmar to western Thailand); and records of this species in China (Xizang, Yunnan, and Guangxi), northern Vietnam, and northern Laos can apply to other named and unnamed species. However, Lyu et al. (2023) provided a detailed account of *Xenophrysparva*, placing it in the *Xenophryslekagulii* group, as well as providing an account and specifically including genetically confirmed records of the species from Chiang Mai (Thailand), Laos and north-western Vietnam.

## Supplementary Material

XML Treatment for Leptobrachium masatakasatoi

XML Treatment for Xenophrys parva

XML Treatment for Xenophrys lancangica

XML Treatment for Quasipaa verrucospinosa

XML Treatment for Amolops compotrix

XML Treatment for Kurixalus odontotarsus

85DDA845-2F1C-5BD1-BA0D-106E55CB5CB0 10.3897/BDJ.12.e122598.suppl1 Supplementary material 1 List of amphibians species Data type Table Brief description List of amphibians species recorded in Vu Quang NP and Ha Tinh Province, Vietnam. File: oo_1030393.doc https://binary.pensoft.net/file/1030393 Vinh Quang Dau, Cuong The Pham, Truong Quang Nguyen, Toan Canh Thai, Anh Dinh Tran, Anh Van Pham
