# Supplementary material for: New records of amphibians from Ha Tinh Province, Vietnam
Source: Biodivers Data J. 2024 May 8;12:e122598. doi: 10.3897/BDJ.12.e122598 (PMC11096727; doi:10.3897/BDJ.12.e122598)
Supplement: Supplementary material 1 — List of amphibians species [file bdj-12-e122598-s001.doc]

**Supplementary file 1**

New records of amphibians from Ha Tinh Province, Vietnam

Vinh Quang Dau, Cuong The Pham, Truong Quang Nguyen, Toan Canh Thai, Anh Dinh Tran, Anh Van Pham

Table. List of amphibians species recorded in Vu Quang NP and Ha Tinh Province, Vietnam (1 = Nguyen et al. (2009), 2 = Dau et al. (2015), 3 = Thai (2017), 4 = Ziegler et al. (2015), 5 = Hoang et al. (2021), 6 = Sheridan et al. (2023), 7 = This study.

| **Name** | **Previous record**  **from Ha Tinh Province** | **Previous record**  **from Vu Quang NP** | **This study** |
| --- | --- | --- | --- |
| **Ichthyophiidae** |  |  |  |
| *Ichthyophis kohtaoensis* Taylor, 1960 | 4 |  |  |
| **Bufonidae** |  |  |  |
| *Ingerophrynus galeatus* (Günther, 1864) | 1, 4 | 1 |  |
| *Duttaphrynus melanostictus* (Schneider, 1799) | 1, 4 | 1 |  |
| **Megophryidae** |  |  |  |
| *Boulenophrys* cf. *parva* (Boulenger, 1893) |  |  | 7 |
| *Leptobrachium chapaense* (Bourret, 1937) | 1, 4 | 1 |  |
| *Leptobrachium masatakasatoi* Matsui, 2013 |  |  | 7 |
| *Leptobrachella aerea* (Rowley, Stuart, Richards, Phimmachak & Sivongxay, 2010) | 4 |  |  |
| *Ophryphryne hansi* (Ohler, 2003) | 1, 4 |  |  |
| *Xenophrys* cf. *maosonensis* (Bourret, 1937) | 4 | 1 |  |
| *Xenophrys lancangica* Lyu, Wang & Wang, 2023 |  |  | 7 |
| **Microhilydae** |  |  |  |
| *Microhyla butleri* Boulenger, 1900 | 1, 4 | 1 |  |
| *Microhyla mukhlesuri* Hasan, Islam, Kuramoto, Kurabayashi & Sumida, 2014 | 1, 4 |  |  |
| *Microhyla pulchra* (Hallowell, 1861) | 1, 4 | 1 |  |
| *Micryletta inornata* (Boulenger, 1890) | 1, 4 |  |  |
| *Nanohyla marmorata* (Bain & Nguyen, 2004) | 1 |  |  |
| *Vietnamophryne vuquangensis* Hoang, Jiang, Nguyen, Orlov, Le, Nguyen, Nguyen, Nguyen, Nguyen & Ziegler, 2021 | 5 |  |  |
| **Dicroglossidae** |  |  |  |
| *Fejervarya limnocharis* (Gravenhorst, 1829) | 1, 4 | 1 |  |
| *Hoplobatrachus chinensis* (Osbeck, 1765) | 1, 4 | 1 |  |
| *Limnonectes bannaensis* Ye, Fei, Xie & Jiang, 2007 | 1, 4 | 1 |  |
| *Limnonectes* cf. *limborgi* (Sclater, 1892) | 4 |  |  |
| *Occidozyga lima* (Gravenhorst, 1829) | 4 |  |  |
| *Phrynoglossus* cf. *martensii* Peters, 1867 | 4 |  |  |
| *Quasipaa* cf. *verrucospinosa* (Bourret, 1937) |  |  | 7 |
| **Ranidae** |  |  |  |
| *Amolops compotrix* (Bain, Stuart, and Orlov, 2006) |  |  | 7 |
| *Amolops cremnobatus* Inger & Kottelat, 1998 | 6 |  |  |
| *Odorrana chloronota* (Günther, 1876) | 1, 4 | 1 |  |
| *Odorrana bacboensis* (Bain, Lathrop, Murphy, Orlov & Ho, 2003) | 1, 4 | 1 |  |
| *Odorrana morafkai* (Bain, Lathrop, Murphy, Orlov & Ho, 2003) | 1 | 1 |  |
| *Odorrana tiannanensis* (Yang and Li, 1980) | 1 | 1 |  |
| *Rana johnsi* Smith, 1921 | 1, 4 | 1 |  |
| *Sylvirana annamitica* Sheridan & Stuart, 2018 | 4 |  |  |
| *Sylvirana guentheri* (Boulenger, 1882) | 1, 4 | 1 |  |
| *Sylvirana maosonensis* (Bourret, 1937) | 1 | 1 |  |
| **Rhacophoridae** |  |  |  |
| *Feihyla vittata* (Boulenger, 1887) | 1, 3, 4 | 1 |  |
| *Kurixalus odontotarsus* (Ye & Fei, 1993) |  |  | 7 |
| *Orixalus ananjevae* (Matsui and Orlov, 2004) |  | 1 |  |
| *Polypedates megacephalus* Hallowell, 1861 | 1, 4 | 1 |  |
| *Polypedates mutus* (Smith, 1940) | 4 | 1 |  |
| *Rhacophorus kio* Ohler & Delorme, 2006 | 1, 4 | 1 |  |
| *Rhacophorus orlovi* Ziegler & Köhler, 2001 | 1, 4 | 1 |  |
| *Theloderma corticale* (Boulenger, 1903) | 2 |  |  |
| *Zhangixalus dennysi* (Blanford, 1881) | 1, 4 | 1 |  |
